# Effects of Four Marine Toxins on Murine Hepatic Biotransformation Enzymes †

**DOI:** 10.3390/toxins18080331

**Published:** 2026-07-30

**Authors:** Joanna Soto de Jesus, Carmen González-Keelan, Peter A. Meléndez, Carmen L. Cadilla, Jasmine Contreras, Braulio D. Jiménez-Vélez

**Affiliations:** 1 Department of Pharmaceutical Sciences, School of Pharmacy, University of Puerto Rico, Medical Sciences Campus, San Juan, PR 00936, USA; joasot13@yahoo.com; 2 Department of Pathology, School of Medicine, University of Puerto Rico, Medical Sciences Campus, San Juan, PR 00936, USA; 3 Department of Biochemistry, School of Medicine, University of Puerto Rico, Medical Sciences Campus, San Juan, PR 00936, USA; melendez.peter@gmail.com (P.A.M.); carmen.cadilla@upr.edu (C.L.C.); 4 School of Medicine, Universidad Central del Caribe, Bayamón, PR 00960, USA; 122jcontreras@uccaribe.edu

**Keywords:** marine harmful algae bloom, hepatic enzymes, maitotoxin-2, ciguatoxin-1, brevetoxin-2, saxitoxin

## Abstract

This study assesses the impact of sublethal levels of four marine toxins ciguatoxin (CTX-1), maitotoxin-2 (MTX-2), saxitoxin (STX) and brevetoxin-2 (BTX-2) on murine hepatic detoxification enzymes expressed in mouse liver. CTX-1, BTX-2, and STX altered hepatic detoxification responses, but their effects were generally more limited or temporally variable than those observed with MTX-2. CTX-1 produced time-dependent changes in cytochromes P450 (CYPs)-associated activities, BTX-2 induced early CYP1A2 and CYP3A11 responses followed by later suppression, and STX reduced CYP1A2 and CYP3A11 while increasing microsomal reductase activities. Of these toxins, MTX-2 exhibited the highest toxicity, notably decreasing key proteins such as CYPs, including CYP1A2 and CYP3A11. CYP enzymes are vital for metabolizing important endogenous and various xenobiotic substances, including therapeutic drugs. After MTX-2 exposure, both CYP1A2 and CYP3A11 levels dropped significantly at 12 h (*p* < 0.0001 for CYP3A11, *p* < 0.0001 for CYP1A2). Histopathological analysis revealed liver damage; however, albumin mRNA levels remained stable post-MTX-2 treatment, indicating that hepatotoxicity was not the sole cause of CYP3A11 reduction. Immunohistochemical analysis displayed uniform CYP3A11 distribution across liver regions after MTX-2 treatment. This suggests that MTX-2 exposure could augment the toxicity of drugs like Aldactone, Erythromycin, and Cyclosporine that utilize this metabolic pathway in humans. This is the first type of research performed of this nature, which could add to our understanding of marine toxin metabolism. These findings provide additional toxicological insights into the effects of four marine toxins on detoxification enzymes, with particular interest in MTX-2 toxicity, and potential implications for the treatment of fish poisoning, including ciguatera fish poisoning.

## 1. Introduction

Organisms are constantly being exposed to numerous types of both natural and anthropogenic environmental stressors. Human activities such as dredging, pollution, coral reef degradation, and eutrophication have been reported to promote harmful algal blooms (HABs), particularly the proliferation of toxin-producing dinoflagellates, thereby increasing the frequency, abundance, and bioavailability of several marine biotoxins [1]. The frequencies and intensities of harmful algal blooms (HABs) seem to be increasing globally; this is primarily due to factors such as the rise in ocean temperatures (eighteen of the warmest years have occurred in the twenty-first century), global climate change which is leading to changes in water circulation patterns, and the exacerbation of coastal eutrophication [2,3,4]. Among the many microalgal species found in nature, including dinoflagellates, approximately 300 are associated with harmful events, and more than 100 are known to produce persistent natural toxins, including marine toxins [5]. The transfer of some marine toxins such as ciguatoxin (CTX-1), maitotoxin (MTX-2), saxitoxin (STX) and brevetoxin (BTX-2) through the food chain can lead to various forms of human poisoning, including ciguatera [6], paralytic shellfish poisoning by the saxitoxin group of toxins [7], and neurotoxic shellfish poisoning by molluscan shellfish [8]. The effects of these marine toxins are global [9], CTX-1 impacting an estimated 50,000 individuals annually in tropical and subtropical regions [10,11]. In addition, global warming has become a major concern because it may contribute to an increase in HABs [12], particularly the proliferation of dinoflagellates.

STX, BTX-2, CTX-1, and MTX-2 are among the most described and studied marine toxins [13]. Paralytic shellfish poisoning is caused by STX and related paralytic shellfish toxins (PSTs), which are primarily produced by several dinoflagellate species [14]. However, freshwater cyanobacteria, macroalgae, and certain marine bacteria have also been reported as alternative sources of PSTs [15,16,17].

STX, BTX-2, and CTX-1 all target voltage-gated Na^+^ channels [18,19,20,21]; however, they act through distinct mechanisms and interact with different receptor sites on the channel. An exception is CTX-1 and BTX-2, which both bind to the same receptor site at receptor site 5 on the α-subunit of the channel [22,23]. CTX-1 and BTX-2 share significant structural similarities, as both belong to the family of ladder-shaped polyether marine toxins; however, they also differ considerably in molecular size, rigidity, polarity, and functional group composition. CTX-1 is generally considered far more toxic and persistent than BTX-2. The greater toxicity of CTX-1 is associated with its stronger binding affinity for sodium channels, increased molecular stability, and prolonged activation of neuronal membranes [24].

BTX-2 acts as an activator. It binds to Site 5, lowering the threshold for activation and causing the channel to open at more negative potentials, leading to persistent activation. CTX-1 causes sustained sodium channel activation and blocks potassium channels, it also binds to Site 5 (like BTX-2). It causes a shift in the voltage-dependency of activation toward more negative potentials, leading to persistent sodium channel activation. STX functions as a pore blocker by binding to neurotoxin Receptor Site 1 on the extracellular surface of the channel. This interaction blocks the influx of sodium ions, thereby preventing the generation of action potentials associated with paralytic shellfish poisoning.

MTX-2 is regarded as one of the most potent non-protein marine toxins identified to date. In contrast to CTX-1 and BTX-2, which primarily act on voltage-gated sodium channels, MTX-2 toxicity is predominantly associated with massive calcium influx into cells and disruption of ion homeostasis. MTX-2 is a highly toxic polar substance produced by the dinoflagellate *Gambierdiscus toxicus* [25,26,27]. Maitotoxin and saxitoxin are highly polar hydrophilic toxins, whereas brevetoxins and ciguatoxins are predominantly lipophilic polyether compounds with strong affinity for biological membranes. MTX-2 is a potent activator of Ca^+^ channels and a potential inhibitor of the Na^+^/K^+^ ATPase in various cell types [28,29,30].

Biotransformation of foreign compounds is a crucial process in the metabolism of numerous toxins [31]. Many xenobiotics including marine toxins require metabolic biotransformation to facilitate their elimination and excretion from biological systems, processes that largely depend on detoxification enzymes. The metabolism of many marine toxins is yet to be fully elucidated and there is currently limited evidence supporting extensive CYP450-mediated biotransformation. Lipophilic marine toxins such as brevetoxins are known substrates of hepatic CYP450-mediated biotransformation, whereas evidence for CYP450 metabolism of saxitoxin, maitotoxin, and ciguatoxins remains limited or incompletely characterized. In addition, marine toxins may disrupt detoxification enzymes and alter the expression of genes involved in biotransformation pathways. To address this knowledge gap and further expand research in this area, we evaluated a selection of detoxification enzyme activities following exposure to four common marine toxins. Understanding the effects of marine toxins and their metabolism in mammalian systems is essential for elucidating the underlying mechanisms, interactions, and modes of action of these unique marine compounds. The objective of this research was to investigate the effects of four structurally and mechanistically distinct marine biotoxins-CTX-1, BTX-2, STX, and MTX-2 on hepatic detoxification systems in mice, with particular emphasis on cytochrome P450 enzymes and other phase I and phase II biotransformation pathways. This research contributes to a more comprehensive understanding of how different marine toxins affect mammalian detoxification systems, thereby advancing current knowledge of their biological and toxicological impacts.

## 2. Results

### 2.1. Effects of Marine Toxins on Detoxification Enzymes

#### 2.1.1. MTX-2

**Figure 1:**
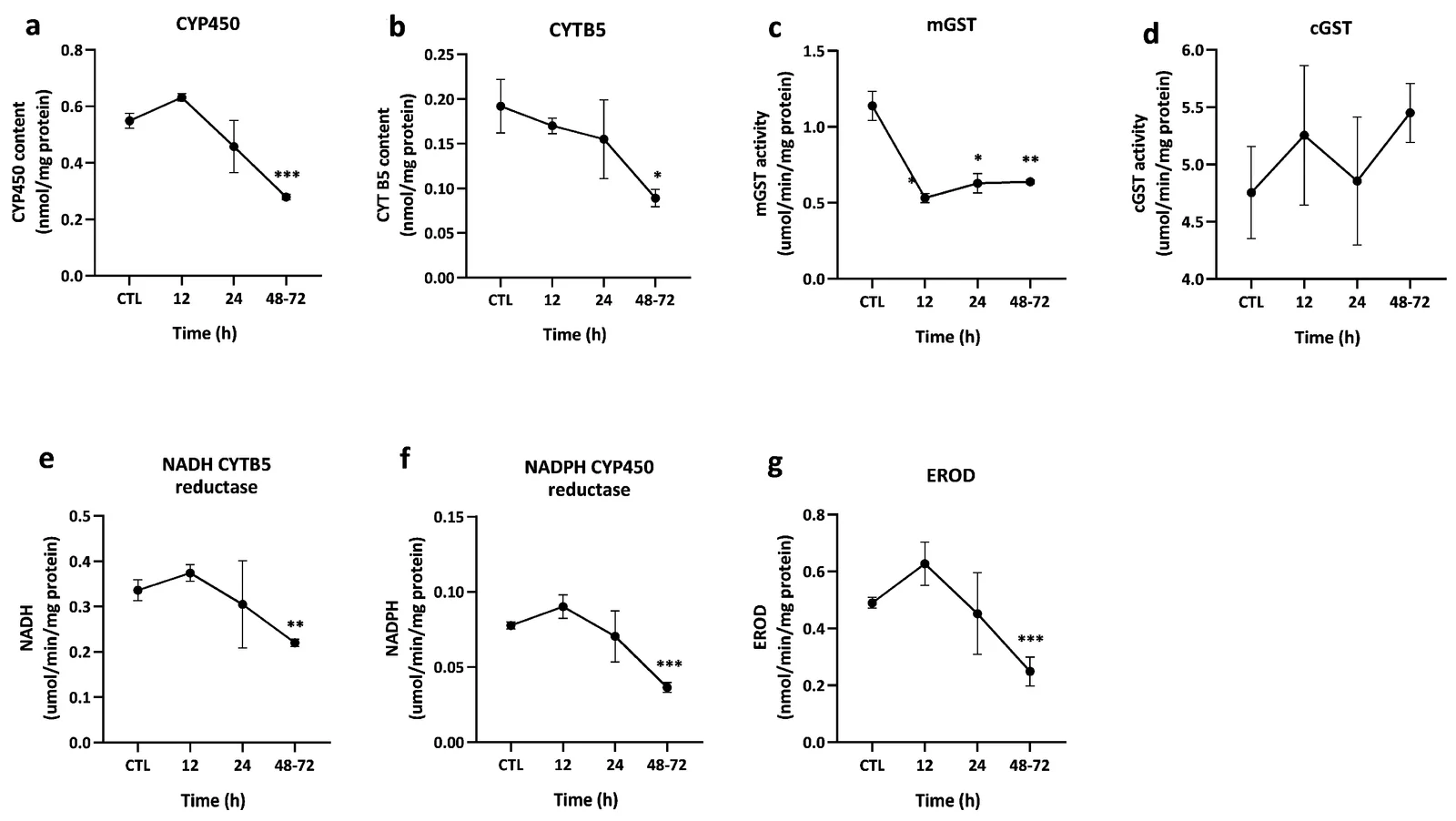
Murine detoxification enzyme concentration and activities after MTX-2 treatment. The levels of Hepatic enzymes cytochrome P450 (**a**) CYP450) and cytochrome b5 (**b**) CYTb5) and activities of microsomal glutathione transferase (**c**) mGST), cytosolic glutathione transferase (**d**) GST), nicotinamide adenine dinucleotide cytochrome b5 (**e**) NADH-CYTb5) reductase, nicotinamide adenine dinucleotide phosphate cytochrome P450 (**f**) NADPH-CYP450) reductase, and ethoxyresorufin-O-deethylase (**g**) EROD) were quantified at various time points (12–72 h) following toxin treatment and compared with its respective controls (CTLs). Each time point represents the mean ± SEM of an independent group exposure. Statistical significance was determined (CI: 95%,) * *p* ≤ 0.05, ** *p*  ≤ 0.01 and *** *p* ≤ 0.001. CTLs (*n* = 3); and each time point (*n* = 2) except for 48–72 (*n* = 3).

**Figure 2:**
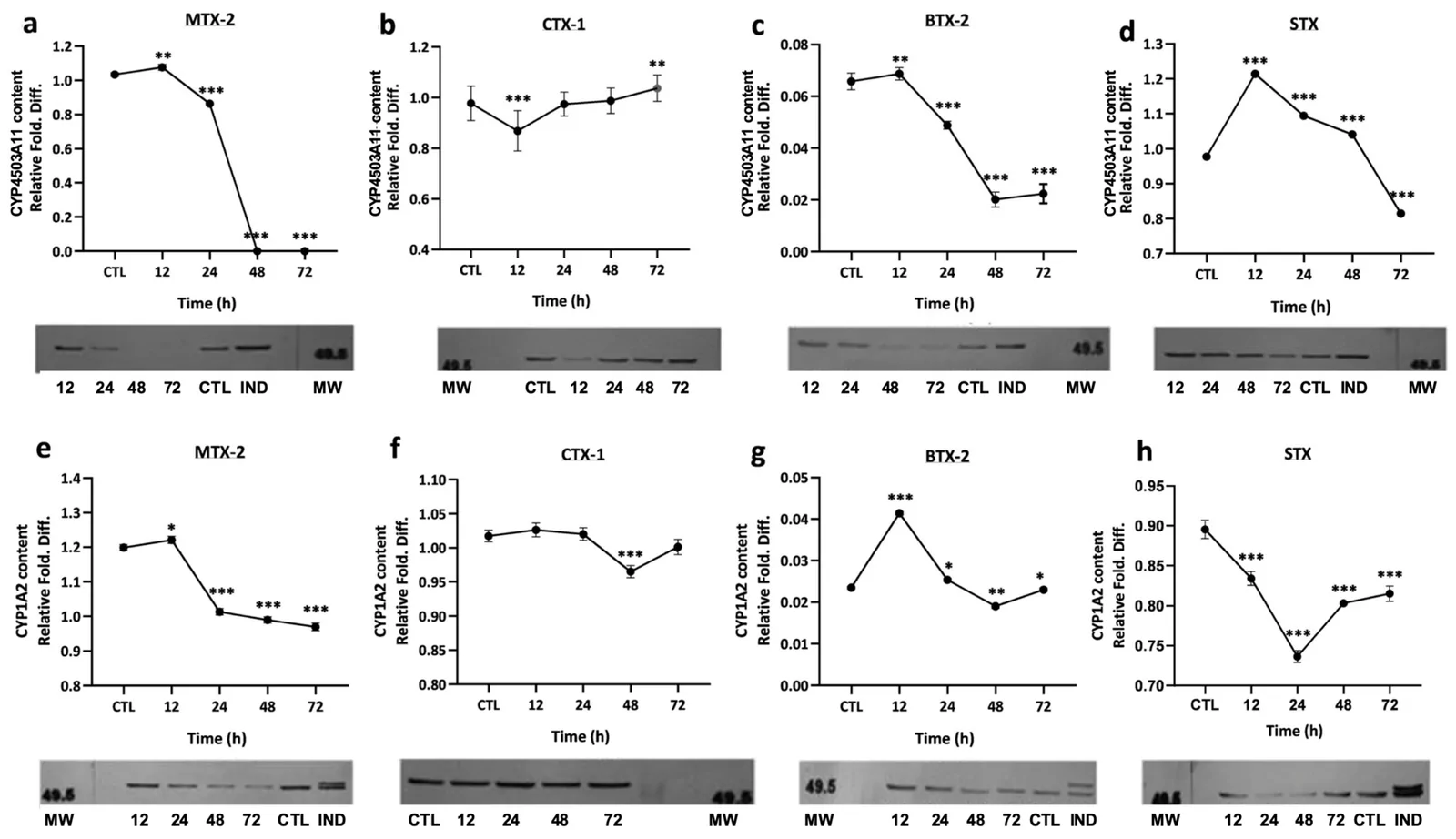
Immunodetection and densitometric analysis of Western blot bands corresponding to (**e**–**h**) cytochrome P450 1A2 (CYP1A2) and (**a**–**d**) cytochrome P450 3A11 (CYP3A11) in mouse liver microsomes following treatment with (**a**,**e**) maitotoxin-2 (MTX-2), (**b**,**f**) ciguatoxin-1 (CTX-1), (**c**,**g**) brevetoxin-2 (BTX-2), or (**d**,**h**) saxitoxin (STX). Monoclonal antibodies 1-12-3 and 2-13-1 were used to detect mouse CYP1A2 and CYP3A11, respectively. Liver microsomal fractions isolated from treated young male mice at the indicated time points were separated by SDS–PAGE using 15 μg of protein per lane. The upper band in the inducer-control (IND) lane corresponds to CYP1A2. Densitometric data are expressed as mean ± SEM (*n* = 2 per time point). Statistical significance was evaluated at the 95% confidence level, with asterisks indicating significant differences relative to the corresponding control: * *p* ≤ 0.05, ** *p*  ≤ 0.01 and *** *p* ≤ 0.001. Data are presented as mean ± SEM (*n* = 2 per time point).

**Figure 3:**
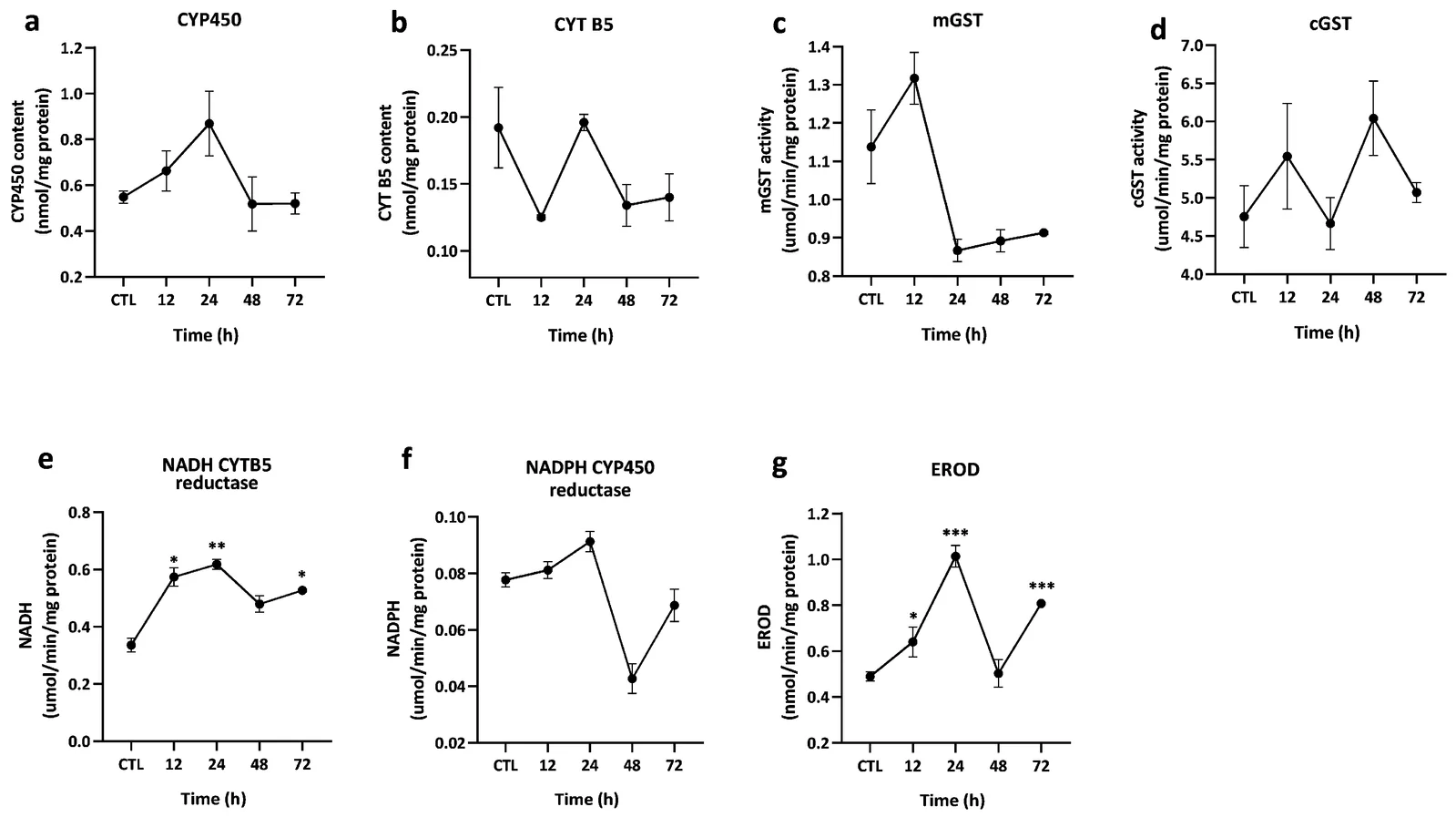
Content and activities of liver detoxification enzymes in mice treated with BTX-2. (**a**) CYP450, and (**b**) CYT b5 content; (**c**) mGST, (**d**) cGST, (**e**) NADH-CYTb5, (**f**) NADPH-CYP450 reductase and (**g**) EROD, enzyme activities were obtained at various time points (12–72 h) after treatment and compared to their respective controls (CTLs). Statistical significance was established (CI: 95%, *p* < 0.05) *p*-values are illustrated with asterisks: * *p* ≤ 0.05, ** *p* ≤ 0.01 and *** *p* ≤ 0.001. All data represent the mean ± SEM, CTLs (*n* = 3) and each time point from independent group exposure (*n* = 2).

**Figure 4:**
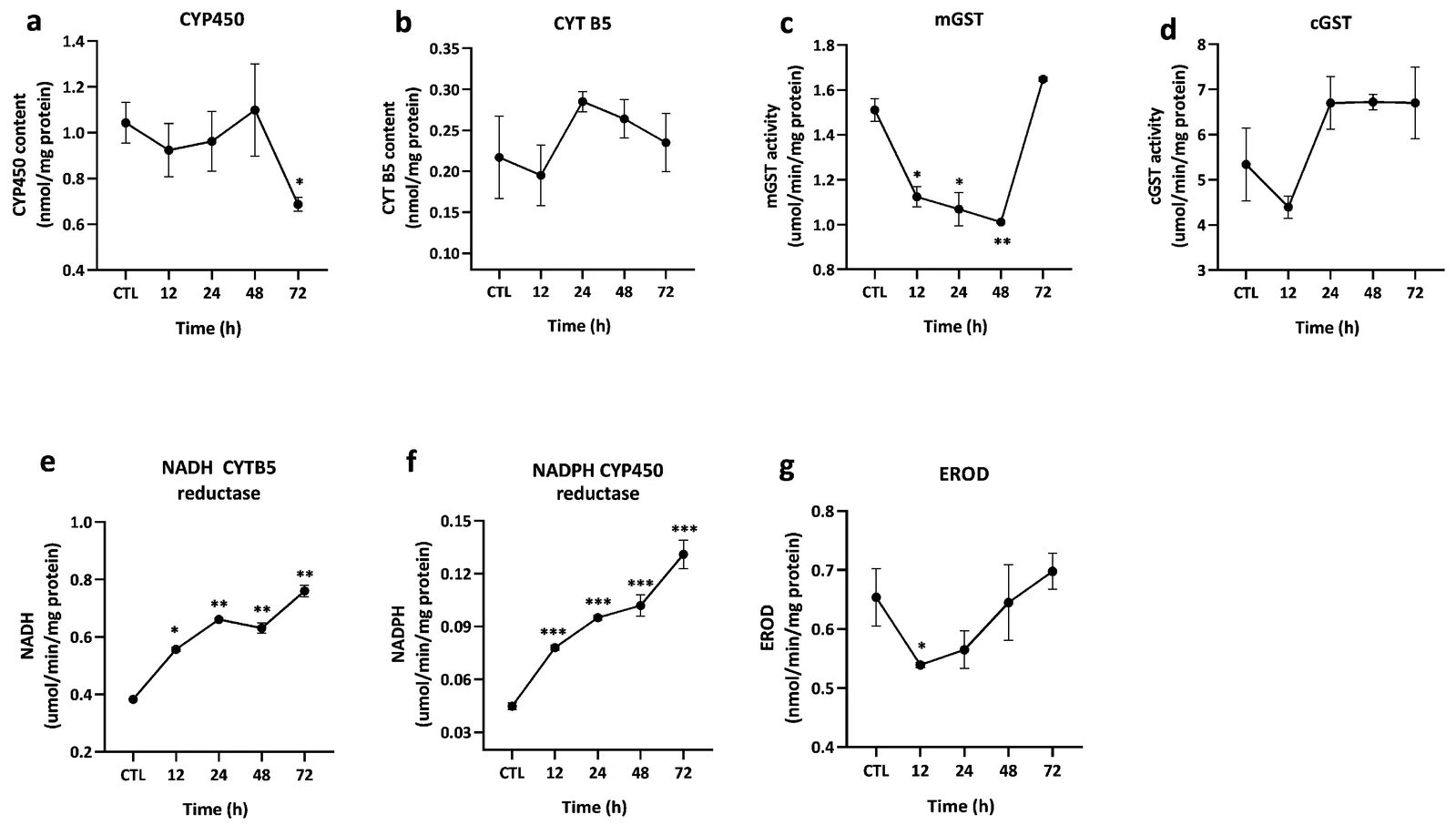
Content and activities of murine hepatic detoxification enzymes following STX treatment. The levels of hepatic enzymes, (**a**) cytochrome P450 (CYP450) and (**b**) cytochrome b5 (CYTb5) and activities of (**c**) microsomal glutathione transferase (mGST), (**d**) cytosolic glutathione transferase (cGST), (**e**) nicotinamide adenine dinucleotide cytochrome b5 (NADH-CYTb5) reductase, (**f**) nicotinamide adenine dinucleotide phosphate cytochrome P450 (NADPH-CYP450) reductase, and (**g**) ethoxyresorufin-O-deethylase (EROD) were quantified at various time points (12–72 h) following toxin treatment and compared with its respective controls (CTLs). Statistical significance was established at 95% confidence interval (*p* < 0.05), with significance levels indicated as * *p* ≤ 0.05, ** *p* ≤ 0.01, and *** *p* ≤ 0.001. Data are presented as mean ± SEM, with (CTLs *n* = 3) and treatment time points comprising *n* = 2.

Hepatic enzyme activities in MTX-2 IP-injected mice from 12 to 72 h showed reductions in most of the parameters evaluated, including NADPH-CYP450 reductase activity (54%, *p* < 0.05; Figure 1f), total CYP450 concentration (49%; Figure 1a), and EROD activity (49%; Figure 1g). This reduction in relative CYP450 content and EROD activity correlates with the Western blot data, in which CYP1A2 and CYP3A11 levels were significantly reduced (Figure 2a,e). MTX-2 was the only toxin that significantly decreased (*p* < 0.05) overall NADH-cytochrome b5 reductase, NADPH-cytochrome P450 reductase, and EROD activity (Figure 1e–g). Alterations in hepatic m-GST activity were among the earliest markers observed following MTX-2 treatment, with significant reductions detected as early as 12 h post-treatment. This marker exhibited an approximately 50% reduction in content as early as 12 h following MTX-2 treatment (Figure 1c). However, cytosolic GST activity in most mice injected with the other marine toxins did not decrease over time (Figure 1d, Figure 3 and Figure 4d). In contrast, cGST activity generally increased over time, suggesting that this enzyme is not markedly affected by the marine toxins evaluated in this study. Among all toxins evaluated, MTX-2 exerted the greatest reduction in the activity of most detoxification enzymes tested. Notably, cGST was one of the earliest biomarkers affected following MTX-2 treatment, displaying an approximately 50% reduction in activity as early as 12 h post-treatment (Figure 1c).

#### 2.1.2. CTX-1

**Figure 5:**
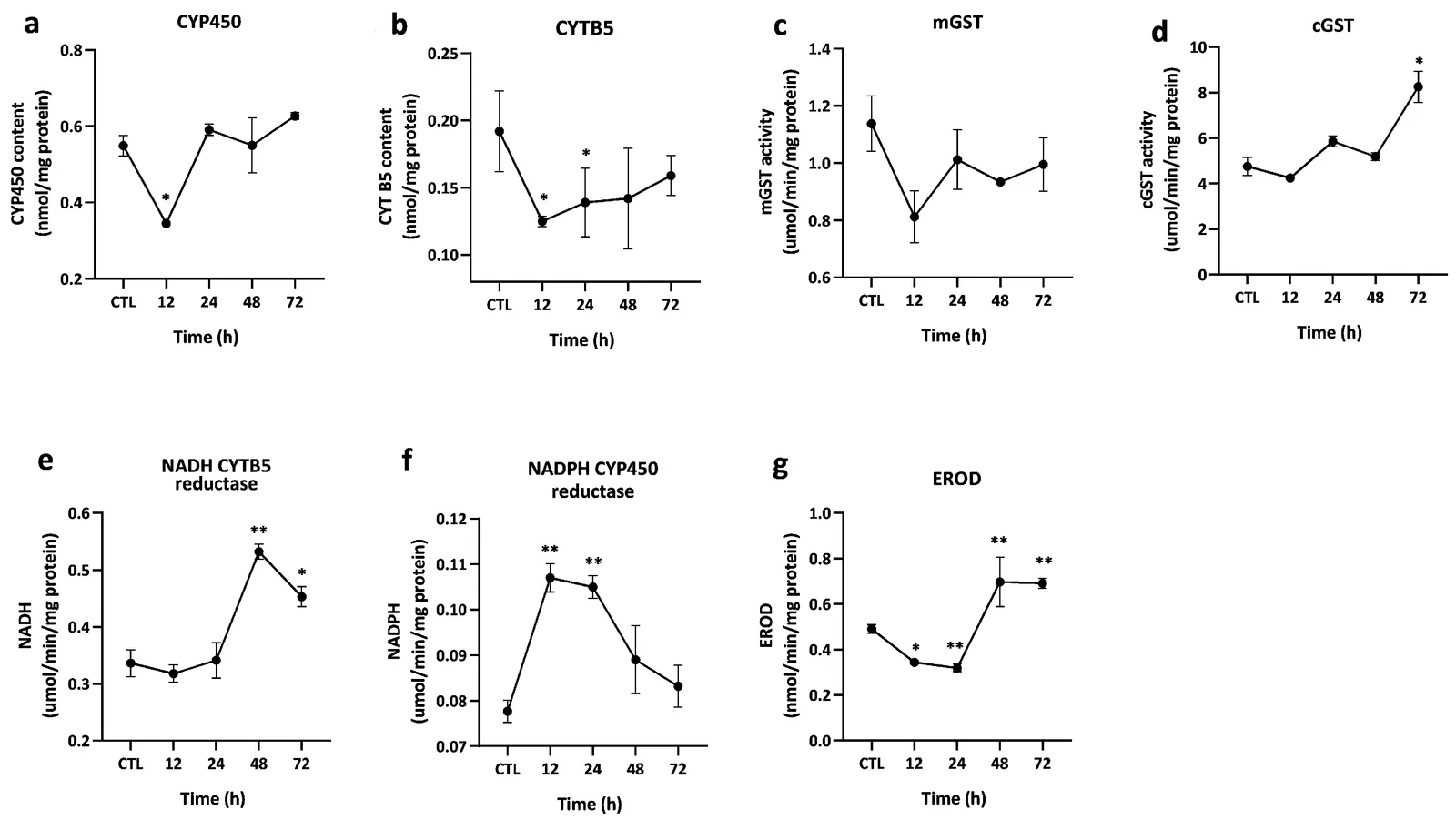
Content and activities of murine detoxification enzymes treated with CTX-1. Levels of hepatic cytochrome P450 (CYP450) (**a**), cytochrome b5 (CYTb5) (**b**); activities of microsomal (mGST) (**c**), and cytosolic (cGST) (**d**) glutathione transferases; NADH-cytochrome b5 (**e**) and NADPH-cytochrome P450 (**f**) reductases, ethoxyresorufin-O-deethylase (EROD) (**g**) were quantified at (12–72 h). Levels and activities of enzymes were compared with their respective controls (CTLs). Statistical significance was established at CI: 95%, * *p* < 0.05, ** *p* ≤ 0.01. Each time point represents the mean ± SEM, of each independent group exposure (*n* = 2). CTLs (*n* = 3).

The main effect observed by CTX-1 was a significant reduction in CYPb5 content and EROD activity at 12 and 24 h, respectively (Figure 5b,g). However, NADPH-CYP450 reductase activity increased at this dose and time point (Figure 5f). No significant changes in mGST, cGST, and NADH CYTb5 reductase activity were observed (Figure 5c–e) at 12–24 h. The reduction in EROD activity coincides with the reduction in total CYP450 at 12 h, which is also observed in the amount of CYP3A11 at the same these time points (Figure 4b). Interestingly, cGST, NADH-CYTb_5_ reductase, and EROD activities increased at 48–72 h. This increase in EROD activity also coincides with an observable increase in CYP1A2 and CYP3A11 protein relative concentration at these time points (Figure 2b,f).

#### 2.1.3. BTX-2

No significant differences were found in most of the murine enzymes tested at different time intervals, except for NADH CYTb_5_ reductase and EROD activity and an apparent increase in total CYP450. This increase in CYP450 and EROD at 12 and 24 h is accompanied by an increase in both CYP3A11 and CYP1A2 (Figure 2c,g). Also seen on NADH CYTb_5_ reductase always increased (Figure 3e) and EROD activity at 24 and 72 h (Figure 3g). Interestingly, BTX-2 was shown to decrease (*p* < 0.05) both CYP3A11, and 1A2 at 48–72h (Figure 2c,g) despite no significant change in total CYP450 (Figure 3a). Most astonishing is the dramatic reduction in MGST and NADPH CYP450 reductase at 48 and 72 h (Figure 3c,f).

#### 2.1.4. STX

No significant differences were observed in total CYP450 levels between 12–48 h or in CYTb5 content at any time point (Figure 4a,b). However, after 48 h, a significant reduction in total CYP450 content was detected. MGST activity was also significantly reduced between 12–48 h but returned to control levels by 72 h (Figure 4c). In addition, EROD activity was reduced at 12 h. Both CYP1A2 and CYP3A11 were significantly reduced after STX exposure (Figure 2d–h). In contrast, NADH-cytochrome b5 reductase and NADPH-cytochrome P450 reductase activities (Figure 4e,f) increased significantly (*p* < 0.05) between 12–72 h. despite CYP1A2 & CYP3A11 reduction. cGST activity initially decreased at 12 h and subsequently increased at later time points; however, these changes were not statistically significant due to high variability.

### 2.2. Quantification of CYP3A11 & CYP1A2 After Toxin Treatments Using Western Blots

A significant reduction in CYP3A11 (~81%) and CYP1A2 (~25%) was observed 24 h following MTX-2 treatment compared with controls (Figure 2a,e). This reduction is consistent with the concomitant decline in total CYP450 concentrations observed between 24–72 h (Figure 1a). In contrast, both CYP3A11 and CYP1A2 exhibited an apparent increase at 12 h post-treatment. However, a subsequent reduction in these enzymes was observed between 48–72 h. Like MTX-2, BTX-2 also induced both CYP3A11 and CYP1A2 at 12 h. Thereafter, BTX-2 inhibited CYP3A11 expression after 12 h, whereas CYP1A2 remained induced between 12–24 h before showing a more moderate reduction at later time points. STX treatment exhibited a response pattern like that observed with MTX-2, characterized by an overall reduction in CYP450 levels at most time points (Figure 2d,f), except for CYP3A11 between 24–48 h. However, unlike MTX-2, STX caused an immediate reduction in CYP1A2 levels following exposure (Figure 2h). MTX-2, BTX-2, and STX all induced CYP3A11expression at 12 h, followed by a reduction in enzyme concentration between 24–72 h, whereas CTX-1 exhibited a different response pattern. CTX-1 treatment caused an early reduction, rather than induction, of CYP3A11 at 12 h, followed by increased CYP3A11 levels at later time points. The most pronounced effect of CTX-1 on CYP1A2 was observed after 48 h, when enzyme levels decreased. MTX-2, BTX-2, and STX showed similar response patterns, with initial CYP3A11 induction followed by enzyme suppression; however, MTX-2 produced the strongest effect, almost eliminating CYP3A11 expression between 48–72 h. A similar but less pronounced effect was observed for CYP1A2, which was less affected overall than CYP3A11.

### 2.3. Slot Blot Densitometric Analyses of mRNA Albumin Content

**Figure 6:**
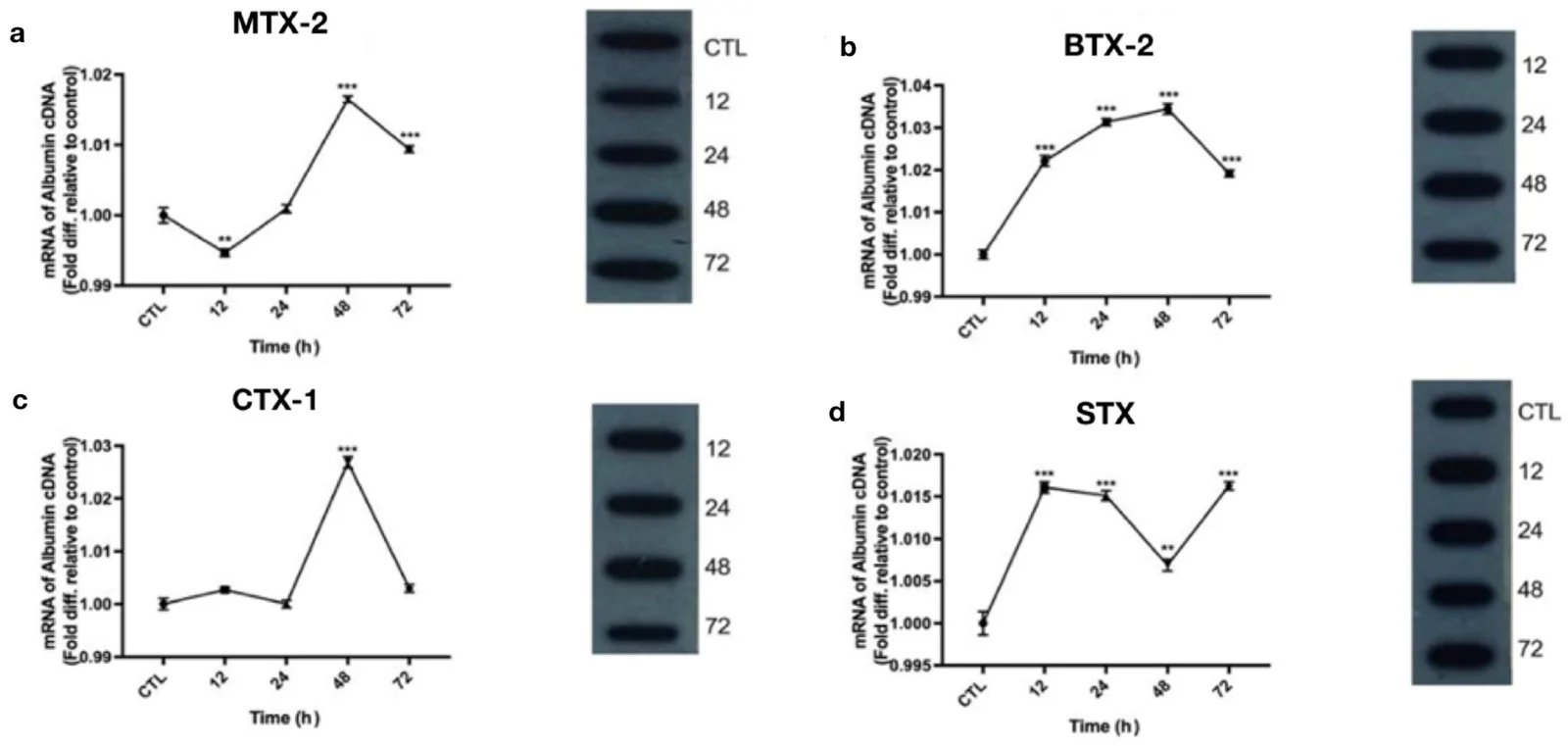
Slot blot and densitometric analysis of mouse liver mRNA using an albumin cDNA probe (pRA57, 250 bp) Following toxin treatment. Liver mRNA was isolated from young male mice treated with (**a**) maitotoxin-2 (MTX-2), (**b**) brevetoxin-2 BTX-2, (**c**) ciguatoxin-1 CTX-1, or (**d**) saxitoxin STX. Control mRNA samples included in the (**a**) MTX-2 treatment groups were also used as controls for the CTX-1 and BTX-2 treatment groups. Statistical significance is indicated as ** *p* ≤ 0.01 and *** *p* ≤ 0.001. Data are presented as mean ± SEM, with (*n* = 2) mice per time point.

No overall reduction in albumin concentration was observed with any of the toxin treatments after 12 to 72 h, except for MTX-2, which was significantly reduced at 12 h. However, this reduction was not permanent, as enzyme levels recovered after 12 h (Figure 6a). In contrast, all toxins increased albumin mRNA levels. Since MTX-2 treatment almost depleted CYP3A11 without significantly reducing albumin levels, these findings support the hypothesis that general hepatotoxicity was not solely responsible for the observed reduction in CYP3A11. Overall, albumin expression levels tended to increase over time after marine toxin exposure.

### 2.4. Histopathology and Immunohistochemistry of Murine Hepatic Tissue

**Figure 7:**
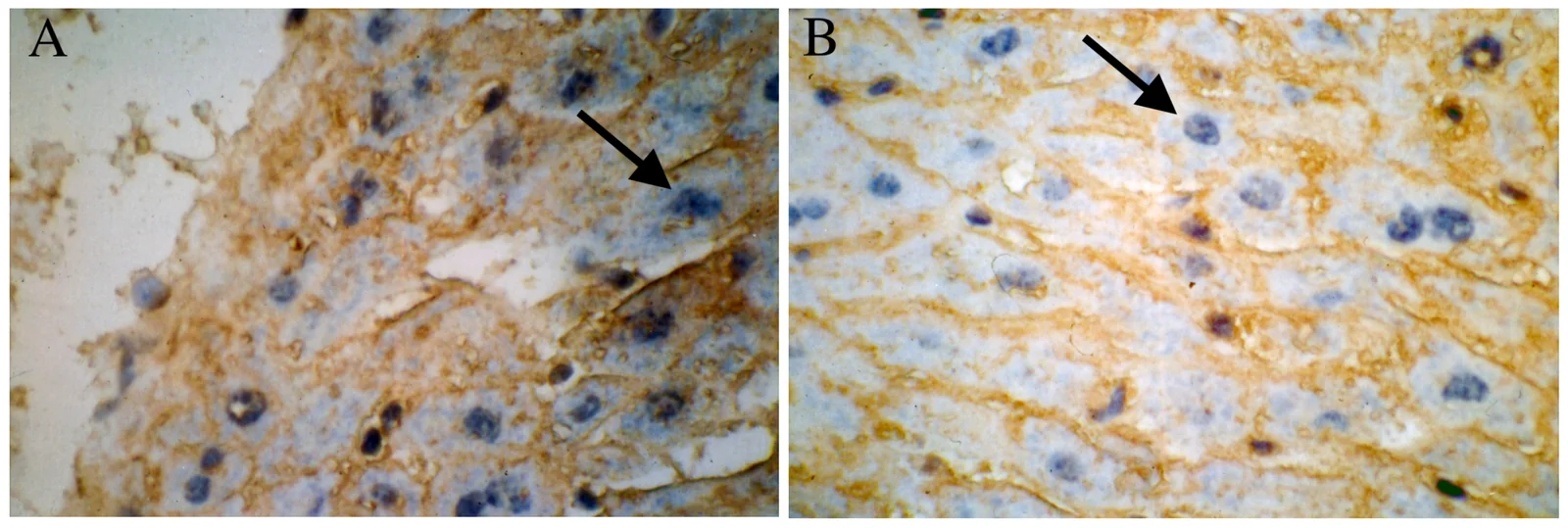
Immunohistochemical localization of cytochrome P450 3A11 (CYP3A11) in different regions of non-treated mouse liver. Hepatic distribution of CYP3A11 was assessed using the monoclonal antibody 2-13-1 directed against CYP3A11. Representative sections show the pericentral region (**A**) and the midzonal region (**B**). Arrows indicate hepatocyte nuclei (dark blue stain) surrounded by light blue cytoplasmic staining representing CYP3A11 immunoreactivity. Under these experimental conditions, CYP3A11 immunoreactivity appeared more intense in the pericentral region than in the midzonal region, consistent with the known zonal distribution of CYP3A enzymes in the liver. The lightly stained or transparent regions surrounding the dark blue nuclei delineate the cytoplasmic boundaries of individual hepatocytes, which measure approximately 10–15 µm in diameter.

**Figure 8:**
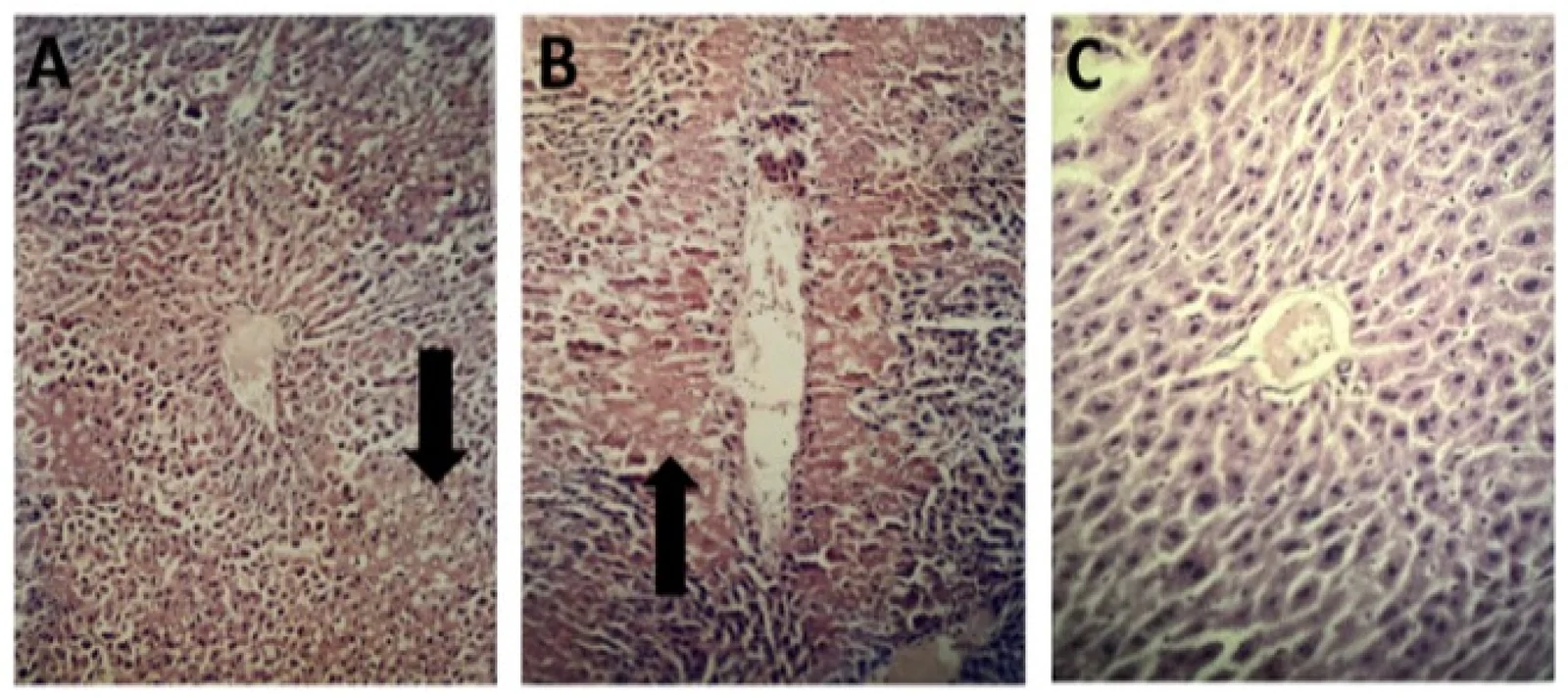
Histological analysis of mouse liver following MTX-2 treatment after 48 h. Arrows indicate necrosis of hepatocytes extending from the pericentral region toward the midzonal region (**A**) and more extensive necrosis surrounding the central vein within the pericentral region (**B**). Loss of cellular cohesion and disappearance of numerous hepatocyte nuclei are evident in the affected areas. Liver sections from control mice treated with 5% Tween-60 saline showed no evidence of necrosis or other histopathological alterations (**C**). Hepatocyte nuclei appear as darkly stained structures surrounded by lightly stained cytoplasm, with individual hepatocytes measuring approximately 10–15 µm in diameter.

In addition to assessing toxin-induced changes in cytochrome P450 expression using cDNA probes and evaluating hepatic detoxification enzyme activities, we performed liver histopathological examinations to determine whether marine toxin exposure produced structural liver damage. Histopathological analysis revealed varying degrees of hepatotoxicity, characterized by microvascular fatty change and hepatic necrosis, following treatment with the four marine toxins (Table 1). MTX-2 produced the most pronounced lesions, with necrosis observed at 12 h (mild) and 48 h (severe), primarily affecting the midzonal and pericentral regions of the liver (Figure 7A and Figure 7B respectively). More limited focal necrosis was also observed following CTX-1 treatment (12 and 24 h) mild necrosis, BTX-2 treatment (72 h) mild, and STX treatment (24 and 48 h) mild. In contrast, histopathological examination of control animals (12, 48–72 h) revealed no evidence of liver necrosis at any of the evaluated time points (Figure 8C).

Hepatic zonation of cytochrome P450 expression has been well documented, with several CYP isoforms, including members of the CYP3A and CYP1A families, exhibiting preferential expression within the pericentral region of the liver lobule [32,33,34,35]. Because MTX-2 produced the greatest hepatotoxicity and the most pronounced reduction in CYP3A11 and CYP1A2 expression, immunohistochemical analysis of CYP3A11 was performed to determine its normal hepatic distribution (Figure 7). Consistent with previous reports, CYP3A11 immunoreactivity was more prominent in the pericentral region than in the midzonal region of non-treated mouse liver. Histopathological examination of MTX-2-treated livers demonstrated that the most severe necrotic lesions were localized around the central vein within the pericentral region (Figure 8B). This spatial relationship between the region of greatest CYP3A11 expression and the region exhibiting the most severe MTX-2-induced injury may explain, at least in part, the marked reduction in CYP3A11 immunoreactivity, the suppression of CYP1A2 expression, and the decrease in total CYP450 content observed following MTX-2 exposure (Figure 2a and Figure 5a,e). Collectively, these findings support an association between MTX-2-induced pericentral hepatotoxicity and suppression of hepatic detoxification enzymes.

## 3. Discussion

The present study demonstrates that marine toxins with distinct—and in some cases overlapping—mechanisms of action produce markedly different patterns of hepatic detoxification enzyme regulation. Although certain toxins share common molecular targets, such as CTX and BTX, which both bind to site 5 of voltage-gated sodium channels, activation of the same primary target does not necessarily result in identical downstream biological responses. Differences in molecular structure, receptor affinity, toxicokinetics, tissue distribution, and activation of intracellular signaling pathways can lead to distinct patterns of gene expression and regulation of hepatic biotransformation enzymes.

Consistent with this concept, CTX-1 and BTX-2 produced relatively similar responses in several hepatic detoxification enzymes (Figure 3 and Figure 5). For example, NADH–cytochrome b5 reductase and EROD activities generally increased following exposure to both toxins. Cytosolic GST activity also increased after treatment with both toxins, although BTX-treated samples exhibited greater variability. In contrast, microsomal GST activity and cytochrome b5 concentrations showed an overall decreasing trend following exposure to both toxins. Despite these similarities, important differences were observed in CYP regulation. CTX-1 and BTX-2 exhibited distinct temporal patterns of CYP3A11 expression, with CTX-1 showing recovery and induction at later time points, whereas BTX-2 produced an initial induction followed by suppression of CYP3A11. CYP1A2 expression was also differentially regulated, with BTX-2 producing a marked transient increase at 12 h. These findings suggest that factors beyond the shared interaction with voltage-gated sodium channels contribute to the differential regulation of hepatic detoxification enzymes by these marine toxins.

A recent (2024) review article on the effects of marine biotoxins states that they can disrupt the body’s metabolic barrier by altering CYP enzyme expression and activity in the liver and intestine [35]. Among the 37 relevant publications identified in this review, most investigated the regulation of CYP450 expression by okadaic acid (OA). In contrast, only one or two studies have investigated the effects of CTX (ciguatoxin) and BTXs (brevetoxins) on CYP450 regulation, whereas MTX and STX have not been addressed in the literature. Only limited studies have examined the interaction between brevetoxins and xenobiotic-metabolizing enzymes (phase I and phase II), although oxidative and conjugative metabolic pathways for brevetoxins have been previously characterized in vitro [36]. They reported that both CYP1A1 and CYP3A1 are involved in BTX-2 metabolism. BTX-2 has also been reported to increase the activity of CYP1A and GST in fish [37]. In vitro studies in rat hepatocytes have further shown that brevetoxin-2 (BTX-2) undergoes metabolism through multiple CYP450 isoforms, including CYP1A2, CYP2A2, CYP2C11, CYP2D1, and CYP3A1 [36]. In addition, previous toxicogenomic analysis of brevetoxin exposure in mice reported limited hepatic detoxification-gene responses, with Cyp4a14 induction observed only in a single treated liver sample [38]. Our findings further support the ability of BTX-2 to alter hepatic detoxification pathways, while additionally demonstrating time-dependent modulation of CYP1A2 and CYP3A11 expression. In the present study, BTX-2 induced both CYP1A2 and CYP3A11 expression at 12 h, followed by a significant reduction in both enzymes after 48 h (Figure 4). These findings are further supported by the induction of EROD activity observed at 12–24 h and again at 72 h following BTX-2 exposure (Figure 5g). This finding correlates with studies performed in fish using BTX-2 [37] where EROD induction was also reported.

CTX-1 studies have included expression profiling in mouse liver, revealing significant alterations in the expression of 27 genes, primarily belonging to the CYP2 and CYP4 families [39]. CTX-1 exposure induced several CYP2 isoforms, including CYP2B10, CYP2B13, CYP2B9, CYP2E1, and CYP2J9, while CYP3A44 expression was inhibited. The current study shows that CTX-1 treatment produced a time-dependent effect on CYP3A11 expression, characterized by an early reduction at 12 h followed by increased expression at 72 h. Likewise, CYP1A2 content was reduced at 48 h but returned to near-basal levels by 72 h. A similar temporal response was observed for total CYP450 content, which decreased at 12 h and recovered to basal levels at 72 h. However, EROD activity exhibited a biphasic pattern, characterized by early suppression at 12–24 h and subsequent induction at 48–72 h, accompanied by increased NADPH-CYP450 reductase and NADH-cytochrome b5 reductase activities, suggesting compensatory modulation of microsomal electron transport and CYP-associated detoxification pathways. Induction of CYP450 enzymes by xenobiotics such as phenobarbital is frequently accompanied by increased synthesis of NADPH-cytochrome P450 reductase and other microsomal electron transport components [40].

The effects of STX and MTX on detoxification enzymes have not been adequately characterized. This may be attributed to the highly polar nature of these marine toxins, although they differ substantially in molecular size and physicochemical properties, which may facilitate their rapid excretion by the organism. Since marine toxins were administered IP in the present study, this route of exposure may have increased the effective retention time and systemic bioavailability of STX and MTX-2, thereby enhancing hepatic exposure to these toxins. Consequently, this may have amplified the modulatory effects of these toxins on detoxification enzyme expression.

Compared with other marine biotoxins, the interaction between STX and CYP450-dependent detoxification pathways remains poorly characterized. Most studies on STX have focused primarily on its neurotoxic effects and voltage-gated sodium channel blockade, while limited information is available regarding its effects on hepatic CYP450 regulation. The importance of the present study lies in the observation that STX exposure modulates CYP1A2 and CYP3A11 expression, alters EROD activity, and induces both NADPH-cytochrome P450 reductase and NADH-cytochrome b5 reductase responses. Thus, this research addresses an important gap in the current literature by providing novel insights into hepatic detoxification responses to STX, the temporal regulation of CYP-associated pathways, and the potential adaptive responses of microsomal detoxification systems.

In the present study, MTX-2, BTX-2, and STX significantly decreased CYP3A11 expression after 12 h of exposure. Notably, MTX-2 exerted the greatest modulatory effect on CYP3A11 among the toxins evaluated. Although the molecular mechanism responsible for this effect was not investigated in the present study, the pronounced effects of MTX-2 may be related to its well-established ability to disrupt intracellular calcium homeostasis through membrane-associated mechanisms. Such disturbances could influence intracellular signaling pathways involved in the regulation of cytochrome P450 enzymes, including CYP3A11. Consistent with these observations, MTX-2 also produced the greatest hepatotoxicity, inducing cellular necrosis in several regions of the mouse liver, with the most severe lesions localized to the pericentral region.

Another plausible mechanism by which MTX-2 may influence CYP3A11 expression involves the transcriptional regulators pregnane X receptor (PXR) and constitutive androstane receptor (CAR), which are the principal regulators of CYP3A11 expression [41,42,43]. However, the involvement of these receptors was not investigated in the present study. Previous studies have demonstrated that Ca^2+^-dependent activation of protein kinase Cα (PKCα) inhibits PXR activity, thereby suppressing the induction of hepatic CYP3A enzymes [44]. Given the established ability of MTX-2 to disrupt intracellular calcium homeostasis, activation of Ca^2+^-dependent signaling pathways could provide a plausible explanation for the reduction in CYP3A11 expression observed in the present study. In support of this hypothesis, calcium-deficient diets have also been reported to induce CYP3A11 expression [45]. Although these observations suggest a potential mechanistic link between MTX-2-induced calcium dysregulation and suppression of CYP3A11, further studies are required to determine whether PXR, CAR, PKCα, or other calcium-dependent signaling pathways directly mediate this response.

In contrast, STX produced the most pronounced reduction in CYP1A2 expression, causing an immediate decrease in enzyme levels following treatment while also altering CYP3A11 expression.

Two possible mechanisms may explain the necrotic effects of MTX-2 on hepatocytes. MTX-2 may exert a direct cytotoxic effect through activation of Ca^2+^ membrane channels, leading to increased intracellular Ca^2+^ concentrations and subsequent cell death pathways [46,47,48,49]. Alternatively, the effects of MTX-2 on the liver may be indirectly mediated through hormonal and neuroendocrine responses. MTX-2 has been reported to stimulate the release of norepinephrine and to induce a rapid increase in intracellular Ca^2+^ levels in hepatocytes [50]. Although MTX-2 exposure resulted in focal necrosis in different regions of the liver, a reduction in albumin mRNA levels was observed only at 12 h following treatment, coinciding with changes in CYP3A11 and CYP1A2 expression (Figure 4a,e). However, at most time points, albumin levels remained largely unchanged, suggesting that overt hepatocellular injury alone is unlikely to account for the observed reductions in CYP3A11 and CYP1A2 expression. MTX-2 also significantly reduced the activities of NADPH–cytochrome P450 reductase and NADH–cytochrome b5 reductase. This suggests that MTX-2 not only affects CYP450 expression but may also impair the microsomal electron transport system required for optimal CYP450 catalytic activity.

MTX-2 exerted the greatest hepatotoxic effect among the toxins evaluated, inducing hepatic necrosis and suppressing the expression of CYP1A2 and CYP3A11. These findings indicate that MTX-2 can substantially alter hepatic detoxification pathways in mice. Because CYP1A2 and CYP3A enzymes are involved in the metabolism of numerous endogenous and exogenous compounds, suppression of these enzymes may have the potential to alter xenobiotic metabolism. However, the effects of MTX-2 on the disposition, efficacy, or toxicity of co-exposed marine toxins, pharmaceuticals, or other xenobiotics were not evaluated in the present study and therefore remain speculative. Further studies are required to determine whether MTX-2-induced suppression of hepatic detoxification pathways results in biologically significant toxicological interactions. Future studies should further investigate the effects of MTX-2 on cytochrome P450 enzymes using molecular approaches, such as DNA microarrays or probe-based assays or RNA sequencing, to evaluate transcriptional responses at the RNA level across a range of toxin concentrations. RNA sequencing is particularly valuable, since it may reveal effects of these toxins on non-coding RNA gene expression. Of particular importance is the mechanistic interplay between MTX-2 exposure, intracellular calcium signaling, and cytochrome P450 regulation.

In vitro studies employing primary hepatocytes could provide valuable insights into how marine toxins modulate specific cytochrome P450 isoforms, particularly regarding calcium-mediated induction or inhibition mechanisms. In addition, the isolation and characterization of genes altered by specific toxin exposures using gene array or RNA sequencing approaches may further advance understanding of the underlying molecular pathways.

Overall, the findings of the present study support the need for further investigations aimed at elucidating the mechanisms of action of marine toxins and their potential health risks associated with ingestion and intoxication.

## 4. Conclusions

1. This study identifies MTX-2 as the most potent hepatotoxin of the four marine toxins examined, inducing liver damage characterized by necrosis in several hepatic regions and a significant downregulation of the cytochrome P450 enzymes CYP3A11 and CYP1A2. To our knowledge, this is the first study to evaluate the effects of this toxin on multiple murine hepatic detoxification enzymes. These effects are likely mediated by calcium-dependent cytotoxic pathways and may involve suppression of the PXR and CAR, key transcriptional regulators of CYP3A11 expression.
2. Unlike BTX-2, STX and CTX, MTX-2 markedly inhibited NADPH–cytochrome P450 reductase and NADH–cytochrome b5 reductase activities, indicating a broader impairment of hepatic detoxification capacity. Notably, these enzymatic disruptions occurred independently of sustained changes in albumin expression, suggesting that MTX-2 effects are not merely a consequence of generalized hepatotoxicity but rather reflect targeted interference with metabolic regulatory pathways.
3. Among the toxins evaluated, MTX-2 produced the greatest suppression of hepatic CYP3A11 and other detoxification enzymes, indicating that it has the potential to alter hepatic biotransformation capacity. However, the effects of this enzyme suppression on the metabolism, pharmacokinetics, or toxicity of concurrently administered pharmaceuticals or other xenobiotics were not investigated in the present study and therefore remain to be determined.

## 5. Materials and Methods

### 5.1. Animals and Toxins

STX and MTX-2 were prepared in 0.9% sodium chloride saline solution containing 5% Tween 60 (*v*/*v*), corresponding to a ratio of 5 parts Tween 60 to 95 parts saline solution. STX and MTX-2 stock solutions were prepared using a 1:1 mixture of methanol and purified water, whereas BTX-2 and CTX-1 were dissolved in a 1:1 mixture of acetonitrile and methanol. The required toxin amounts were aliquoted from stock solutions, air-dried, and subsequently reconstituted in either 0.9% sodium chloride saline solution (Baxter, Deerfield, IL, USA) containing 5% Tween 60, used as the vehicle for STX and MTX-2, or corn oil, used as the vehicle for BTX-2 and CTX-1.

CTX-1 and MTX-2 were generously provided by Dr. Richard J. Lewis (Marine Fisheries, Canberra, Australia), whose laboratory originally isolated and analytically characterized these marine toxins [51,52]. MTX-2 was reported to be purified to homogeneity by high-performance liquid chromatography, and both CTX-1 and MTX-2 were subsequently characterized by ion spray mass spectrometry in the original studies [51,52]. BTX-2 and STX were provided by Dr. Mark A. Poli (U.S. Army Medical Research Institute of Infectious Diseases, MD). The toxins were supplied as purified, pre-weighed preparations by the collaborators. Certificates of analysis, numerical purity values, and contaminant profiles for the specific toxin lots used in the present study were not available, and no independent analytical verification of toxin identity or purity was performed in our laboratory. This is acknowledged as a limitation of the study. Nevertheless, the biological responses observed are consistent with the established pharmacological activities of these marine toxins and with independent studies reporting modulation of hepatic detoxification enzymes and cytochrome P450 pathways following exposure to purified marine toxins.

A total of 62 (40 gm) male Swiss Webster mice (CFW(SW)BR) were purchased from The Jackson Laboratory (Sacramento, CA, USA). Animals were identified using a marker pen ID on the base of their tail. Mice were intraperitoneally (IP) injected with toxins dissolved in 0.9% sodium chloride saline solution containing 5% Tween 60 (5:95, *v*/*v* Tween 60: saline) for STX and MTX-2. Control animals received the corresponding vehicle alone. BTX-2 and CTX-1 were reconstituted in corn oil, and control animals received corn oil alone. Two mice were treated at each time point (12, 24, 48 & 72 h) with each toxin.

The administered doses of BTX-2 and STX were selected as fractions of the reported LD_50_ values for each toxin (STX and BTX-2, ¼ LD_50_; CTX-1, ⅓ LD_50_; and MTX-2, ⅙ LD_50_ [53,54,55,56]. The doses administered were; CTX-1 (83.33 ng/kg), STX (1.25 µg/kg), BTX-2 (3.23 µg/kg), and MTX-2 (31.7 ng/kg). These sublethal doses were chosen to minimize paralysis, severe distress, or mortality, while allowing assessment of toxin-induced alterations in hepatic detoxification pathways.

Although the primary objective of this study was to investigate the molecular and biochemical effects of marine toxins on hepatic detoxification enzymes rather than to characterize behavioral or clinical manifestations, the animals were routinely monitored throughout the experimental period for general health and signs of toxicity following toxin administration. Formal behavioral assessments were not established as experimental endpoints; therefore, clinical observations were not prospectively recorded using standardized or quantitative scoring systems. Nevertheless, qualitative observations made during the preliminary dose-selection experiments are reported here to provide additional context for the selection of the final experimental doses.

Administration of MTX-2 at one-quarter of the reported LD_50_ resulted in the death of two mice within 24 h. Prior to death, both animals appeared disoriented and exhibited slight elevation of the hind limbs. Consequently, the MTX-2 dose was reduced to one-eighth of the reported LD_50_, at which no apparent behavioral abnormalities were observed, and the final experiments were conducted using one-sixth of the reported LD_50_.

Mice receiving CTX-1 at one-half of the reported LD_50_ exhibited piloerection and reduced spontaneous locomotor activity. In contrast, mice treated with STX at one-half of the reported LD_50_ remained active and did not exhibit obvious behavioral abnormalities. Similarly, mice treated with BTX-2 at doses approaching the reported LD_50_, including animals receiving daily injections for three consecutive days during dose optimization, remained alert and active, with no apparent alterations in locomotor behavior.

These observations were used solely to establish sublethal doses appropriate for the biochemical and molecular studies and should be interpreted as qualitative clinical observations rather than formal behavioral endpoints.

The objective of the present study was to perform an exploratory time-course investigation of the effects of four structurally distinct marine toxins on multiple hepatic detoxification endpoints, including total CYP450 content, individual CYP isoforms, enzyme activities, mRNA expression, histopathology, and immunohistochemistry. Because of the limited availability of several purified marine toxins, ethical considerations regarding animal use, and the large number of experimental groups required (four toxins evaluated at four independent time points together with their corresponding controls), the study was designed using the minimum number of animals necessary to identify temporal trends and generate mechanistic hypotheses.

Although the small sample size (*n* = 2 per each time point) limits statistical power, the major findings were internally consistent across multiple independent endpoints. MTX-2 consistently produced marked reductions in CYP3A11 and CYP1A2 protein expression, decreased total CYP450 content, altered detoxification enzyme activities, and induced characteristic histopathological changes in the liver. The concordance among these independent biochemical, molecular, and histological measurements increases confidence that the observed effects represent biologically meaningful responses rather than random variation between individual animals.

Although several studies have indicated that STX is rapidly eliminated because it is a highly water-soluble toxin, the objective of the present study was to evaluate the temporal effects of acute toxin exposure on hepatic detoxification pathways over a broader post-exposure period (12, 24, 48 and 72 h). IP administration was selected to provide a controlled and reproducible route of systemic exposure for all toxins evaluated. Importantly, the duration of toxin-induced changes in gene and protein expression does not necessarily parallel toxin elimination, as downstream cellular signaling, transcriptional regulation, and protein turnover may persist after the toxin has been cleared. Indeed, the present study demonstrated significant alterations in several hepatic detoxification enzymes at the later time points (24–72 h), indicating that the molecular consequences of acute toxin exposure extend beyond the expected period of toxin elimination. Therefore, the time point profile of 12, 24, 48 and 72 h observation period was selected to characterize the complete temporal profile of hepatic detoxification enzyme regulation following acute exposure to marine toxins.

**Figure 9:**
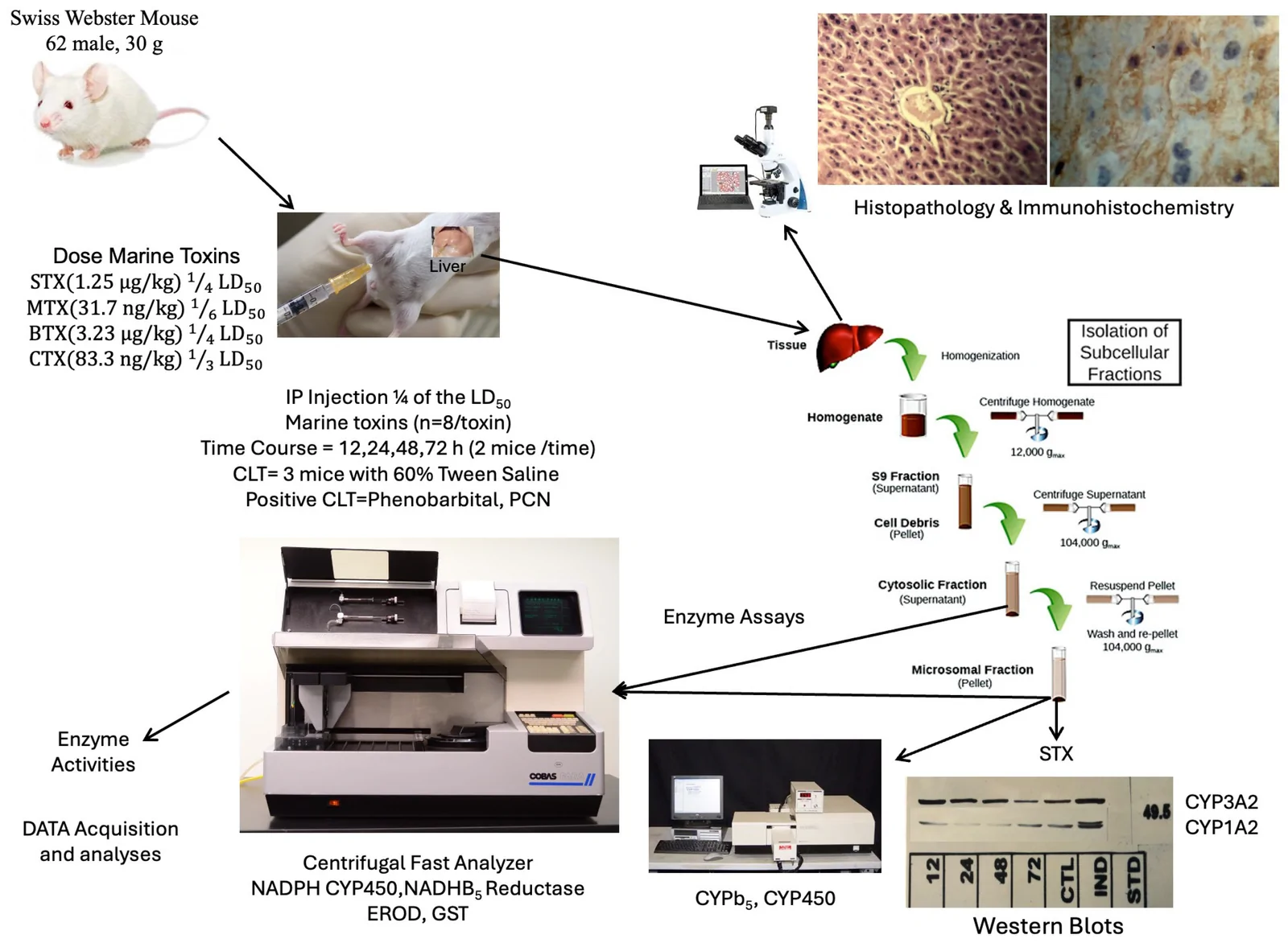
Graphical experimental design. Livers collected from Swiss Webster mice treated by intraperitoneal (IP) injection with sublethal doses of marine toxins, together with control animals administered 5% Tween 60 in saline, were processed for histological and immunohistochemical analyses. Cytosolic and microsomal fractions were isolated for enzymatic assays, and the microsomal fractions were additionally used for Western blot analysis of cytochrome P450 proteins. Arrows indicate the experimental workflow, including the allocation of liver tissue for histopathology and immunohistochemistry and the preparation of microsomal and cytosolic fractions for Western blotting and enzyme activity measurements.

Mice assigned to each toxin treatment group were euthanized by cervical dislocation in accordance with the AVMA Guidelines for the Euthanasia of Animals (2020) at 12, 24, 48 or 72 h following IP injection [57]. Livers were immediately excised, rinsed in homogenization buffer, blotted dry, snap-frozen in liquid nitrogen, and stored for subsequent analyses (Figure 9).

Vehicle-treated controls were included for each toxin class (0.9% saline containing 5% Tween-60 for STX and MTX-2; corn oil for BTX-2 and CTX-1). In addition, three naïve mice per group were maintained without injection and served as secondary controls to assess any effects associated with the injection procedure or vehicle administration. No significant differences were observed between naïve and vehicle-treated control animals; therefore, subsequent comparisons were performed against the respective vehicle controls.

All mice were fed twice daily during designated 2 h periods in the morning and afternoon to reduce hepatic lipid content and facilitate the isolation of liver microsomes.

### 5.2. Sample Preparation and Enzyme Analyses

Liver microsomes were isolated using previously reported procedures [58,59]. Cytosolic and microsomal fractions obtained were stored in liquid nitrogen (–196 °C) until further analysis. Replicate sets of protein concentrations and enzyme analyses were performed on both hepatic microsomal and cytosolic fractions using the Bradford Method [60] adapted for the COBAS FARA II Centrifugal fast analyzer (Roche Diagnostics, Basel, Switzerland). CYP450 and CYTb5 levels were determined from their characteristic oxidized and reduced spectra using a Shimadzu UV-2101PC spectrophotometer (Shimadzu Corporation, Columbia, MD, USA). CYTb5 and CYP450 concentrations were quantified as previously described [61], and results were expressed as nmol cytochrome/mg microsomal protein. Electron transfer enzymes, including NADH-CYTb5 reductase and NADPH-CYP450 reductase, were assayed spectrophotometrically as described [62,63]. Glutathione S-transferase (GST) activity was measured as previously described [64] and ethoxyresorufin O-deethylase (EROD) activity was determined using established methods [63,65]. All spectrophotometric assays were adapted and validated for use with the centrifugal COBAS FARA II auto-analyzer.

### 5.3. Western Blots

SDS-PAGE gel electrophoretic analyses on the microsomal and cytosolic protein fractions were performed as previously described [66,67]. Pre-stained SDS-PAGE standards (5–245 kDa; Bio-Rad, Hercules, CA, USA) were used to compare the relative mobility and to establish protein molecular weights. Proteins separated by SDS-PAGE were transferred onto nitrocellulose membranes for Western blot analysis using the previously described method [68].

Monoclonal antibodies against rat CYP450 isoforms, known to cross-react with mouse CYP450s (P-450IA2, P-4502E1, P-4502B1/2, and P-4503A1/2), were evaluated for their reactivity with mouse CYP450 proteins. All antibodies were generously provided by Dr. Harry Gelboin at the National Cancer Institute [69]. Briefly, membranes were blocked using a 3% non-fat dry milk solution in IX phosphate-buffered saline (PBS), followed by incubation with primary antibodies at 500 µg/mL for 2 h, followed by incubation with an alkaline phosphatase (AP)-conjugated goat anti-mouse secondary antibody (Bio-Rad) at a 1:1000 IgG dilution for at least 1 h. After washing the membranes with 1x PBS and PBS containing 0.05% Tween-20, they were incubated with an AP-conjugated goat anti-mouse antibody at a 1:1000 dilution for 1 h, followed with additional washes with PBS and PBS-0.05% Tween-20. Membranes were then transferred to AP color developer solution containing 1 part nitro blue tetrazolium substrate, 1 part bromochloroindolyl phosphate (both in 70% dimethylformamide) and 100 parts of 0.10 M carbonate buffer pH 9.8 (Sigma, Burbank, CA, USA), incubated for ~10 min, stopping the reaction by washing them with distilled water followed by scanning in a BioRad Imaging Densitometer GS-670.

### 5.4. mRNA Analysis by Slot Blotting

Nitrocellulose or nylon membranes were soaked in deionized water for approximately 5 min, then in 10× SSC for approximately. 5 min. The Slot Blotter was assembled and prewet Whatman filters were laid under the membrane. Bubbles were removed, a slight vacuum was applied, and samples were added. Total RNA samples (~5 µg) were diluted with 20× SSC to a final concentration of 10–15× SSC and an appropriate volume for loading onto a slot blot (100–300 µL). Samples were heated at 70 °C for 5 min., then placed on ice for 1–2 min. prior to loading onto a slot blot. A light vacuum was present during the slotting process. When all the samples were aspirated, a rinse with 10× SSC was performed. The membrane was removed, orientation marks were made, and it was allowed to air dry, followed by fixation in a vacuum oven at 80 °C for 30–45 min. The membrane was then hybridized at 50 °C with the appropriate probes, including a P4503A1 probe (which recognizes P4503A2), kindly provided by Dr. Philip Guzelian (University of Colorado), and an albumin cDNA probe (pRA57). Both probes were radiolabeled with [^32^P] dCTP using the random-primer method [70]. After hybridization, membranes were washed with 1× SSC and exposed to X-ray film, followed by densitometric analyses of the detected signals for the P4503A and albumin mRNAs.

### 5.5. Densitometric Analysis

Densitometric analyses were performed using the imager software and the data obtained from Western blots of mouse liver microsomal proteins, CYP1A2, and CYP3A11. A CYP1A2 and CYP3A11 densitometric control was obtained for each time course (12–72 h) experiment. The data generated the Western blot CYP1A2 and CYP3A11 densitometric mean control value (*n* = 9). This densitometric control value was then compared against the densitometric mean values obtained for CYP1A2 and CYP3A11 at each time course of toxin treatment (STX, CTX-1, MTX-2, and BTX-2). No significant differences were observed between the 48-h control and the control time points of 12, 24, and 72 h. The densitometric data from three Western blots were used to determine the relative average density for CYP1A2 and CYP3A11 protein control. These were compared with the relative densitometric value of CYP1A2 and CYP3A11 at each toxin treatment time interval. Hence, the combined mean of each CYP1A2 and CYP3A11 time point was compared to the combined mean of CYP1A2 and CYP3A11 controls. The final relative densities were graphed and expressed as a fold difference from the average control group, and each of their standard error of the mean (SEM) was determined.

### 5.6. Histopathology and Immunohistochemistry

After sublethal exposure to toxins, histological analyses were performed on several liver samples. Tissue sections were obtained and stained for nucleus distinction using the Harris’ Hematoxylin and Eosin method [71]. Histochemical analyses on CYP3A11 enzymes were performed using the DAKO LSAB kit (Dako Corporation, Carpinteria, CA, USA) [72,73]. Briefly, exposure to primary antibodies were performed at 500 mg/mL for two h and reacted with an AP-conjugated goat-anti-mouse secondary antibody (Bio-Rad) at a 1:1000 dilution of IgG for at least 1 h.

### 5.7. Statistical Analysis

The biochemical response data were organized into two major groups (*n* = 2). The first group included animals evaluated at 12 h and 24 h, whereas the second group included animals evaluated between 48–72 h. Each experimental group was compared with its respective control group (*n* = 3). All enzyme activities and enzyme concentrations were expressed as mean ± SEM. Nonparametric paired and unpaired *t*-tests were used to compare differences among treatments (using Prism software version 11.02). These nonparametric analyses do not assume a normal distribution of the observations and were considered the most appropriate statistical approach for the present data due to the limited number of observations available for densitometric analyses of CYP3A11 and CYP1A2.

## Figures and Tables

**Table 1:** Histopathological alterations observed in mouse liver following exposure to marine toxins and their respective vehicle-treated controls. MTX-2 = maitotoxin-2; CTX-1 = ciguatoxin-1; BTX-2 = brevetoxin-2; STX = saxitoxin; Controls = CLTs. Numbers after each toxin indicate the post-exposure time points (12–72 h). The most severe histopathological alterations were observed 48 h after MTX-2 treatment.

TREATMENT	MICROVASCULAR FATTY CHANGES	MIDZONAL NECROSIS
**TWEEN SALINE CONTROL 48–72 h**	YES	NONE
MTX-12	NONE	MILD
MTX-24	YES	SEVERE
MTX-48	YES	MILD
CTX-12	YES	MILD
CTX-24	YES	NONE
CTX-48	YES	NONE
CTX-72	NONE	NONE
BTX-12	YES	NONE
BTX-24	NONE	NONE
BTX-48	NONE	NONE
BTX-72	NONE	MILD
**CTLs SALINE 48 h**	NONE	NONE
STX-12	YES	NONE
STX-24	YES	MILD
STX-48	YES	MILD
STX-72	NONE	NONE

## Data Availability

The original contributions presented in this study are included in the article. Further inquiries can be directed to the corresponding author.
